# Promoting Self-Regulated Social Media Use on Smartphones With a Mobile Intervention App (Wellspent): Randomized Controlled Trial

**DOI:** 10.2196/56824

**Published:** 2026-04-08

**Authors:** Lea Mertens, Lina Christin Brockmeier, Christina Roitzheim, Theda Radtke, Tilman Dingler, Jan Keller

**Affiliations:** 1 Faculty of Psychology and Neuroscience, Maastricht University, Maastricht, The Netherlands; 2 Department of Education and Psychology, Freie Universität Berlin, Habelschwerdter Allee 45, Berlin, 14195, Germany, 49 03083854906; 3 School of Computing and Information Systems, University of Melbourne, Melbourne, Australia; 4 Wellspent GmbH, Berlin, Germany; 5 Health Psychology and Applied Diagnostics, University of Wuppertal, Wuppertal, Germany; 6 Department of Sustainable Design Engineering, Delft University of Technology, Delft, The Netherlands; 7 Department of Psychology, Heidelberg University, Heidelberg, Germany

**Keywords:** app usage, computer use, digital self-control tool, intervention app, mixed model, nudging, online survey, problematic social media use, randomized controlled trial, screen time, self-efficacy, smartphone use, social media, technology use, well being

## Abstract

**Background:**

Problematic social media use has been linked to reduced well-being and impulse control difficulties. While digital self-control apps show potential for reducing general app usage, they often lack customization, leading to limited effectiveness and increased user resistance. Their impact on problematic social media use remains uncertain.

**Objective:**

This study evaluates the effectiveness of the Wellspent app, a customizable mobile intervention app designed to promote self-regulated social media use by targeting user-defined problematic app use and offering tailored behavioral nudges.

**Methods:**

In a 3-week randomized controlled trial, 70 iPhone users (mean age 26.2, SD 5.6 years; 47/70, 67% female), regularly using at least 1 social media app, were randomly assigned to an intervention (n=35) or control group (n=35). The intervention group received personalized full-screen reminders with the option to quit or continue social media app use whenever an app session exceeded a self-defined time limit. Participants completed weekly online surveys measuring problematic social media use, problematic smartphone use, self-efficacy, and daily screen time on their most problematic app. Linear mixed models tested intervention effects.

**Results:**

While no significant reduction in problematic social media use or increase in self-efficacy was observed, the intervention group showed a significant reduction in daily screen time on their most problematic app by approximately 29 minutes (estimate=−29.35, SE 6.84, 95% CI −42.79 to –15.99; *P*<.001), and a significant decrease in perceived problematic smartphone use (estimate=−0.46, SE 0.18, 95% CI −0.80 to –0.11; *P*=.01).

**Conclusions:**

The Wellspent app demonstrated short-term efficacy in reducing problematic smartphone use. By allowing users to tailor interventions to their personal goals, the app shows promise as a self-directed tool to support healthier digital habits. Further research should explore long-term effects and feature-specific impacts.

## Introduction

### Overview

In 2022, nearly 60% of the world’s population spent an average of 2 hours and 27 minutes per day on social media platforms [1]. While these platforms offer various positive benefits, like providing a sense of closeness or inspiration [2], they also possess [1] features that can foster habitual engagement and contribute to cycles of instant gratification, thereby potentially impeding the pursuit of long-term goals [3].

This duality has spurred an expanding body of research examining the complex relationship between social media use and well-being, highlighting the importance of individual, contextual, and content-related factors in shaping its impact [4-9]. Although findings on the relationship between total time spent on social media and well-being remain inconsistent [7,10], problematic social media use, characterized by excessive, compulsive, or poorly regulated engagement that disrupts daily functioning, has been consistently associated with reduced well-being [7,11,12]. In particular, problematic social media use has been associated with adverse health outcomes, including reduced life satisfaction, increased symptoms of depression, loneliness, and stress [11], as well as lower levels of physical activity [9] and decreased productivity [13]. A meta-analysis by Cheng et al [14] further illustrates the global relevance of this issue, estimating that the prevalence of problematic social media use among adults and adolescents reaches up to 31% in the most affected countries.

### Healthy and Problematic Social Media Use

A healthy balance between connection and disconnection is a key aspect of “digital well-being,” which has been defined as an “optimal balance between the benefits and drawbacks obtained from mobile connectivity” that is achieved “when experiencing maximal controlled pleasure and functional support, together with minimal loss of control and functional impairment” [15]. Problematic social media use, on the other hand, is characterized by a compulsive pattern of social media use that interferes with daily functioning [16]. It displays elements of addictive behaviors, including cognitive salience, mood modification, tolerance, withdrawal, conflict, and relapse [17,18].

The Integrative Model of Mobile Media Use and Need Experiences (IM³UNE model) highlights self-control—the ability to override or inhibit immediate desires in favor of goal-directed behavior [19]—as an important factor in the relationship between mobile connectivity and digital well-being. High levels of self-control protect users from excessive or habitual social media use [20], while deficits in self-control are closely linked to problematic social media use [21].

To better understand self-control in both digital and nondigital environments, research has drawn on the dual-process model. This framework posits 2 modes of information processing: an automatic, impulsive, and unconscious process (System 1), and a reflective, deliberate, and conscious process (System 2) [22]. In the context of problematic social media use, System 1 reflects the habitual, cue-driven use of social media, which is frequently associated with adverse well-being outcomes [23,24].

Specific design features of social media platforms, often referred to as “dark patterns,” are deliberately constructed to exploit System 1 processing and maximize user engagement [25]. For instance, infinite scrolling mechanisms create an illusion of endless content and contribute to compulsive engagement by removing natural stopping cues [26]. Once immersed in these apps, disengaging becomes increasingly difficult, as System 2, despite recognizing the negative consequences of such use, often fails to override automatic behavior. This interplay between automatic engagement and diminished self-regulation underscores the need for interventions that facilitate reflective, intentional technology use. As a result, digital self-control tools have emerged to help individuals regulate their social media use and regain control over their behavior [27].

### Digital Self-Control Tools

Digital self-control tools aim to prompt System 2–driven behavior to support users in regaining self-control over their smartphone use. These tools, often informed by interventionist theories like nudge theory [28], guide users’ choices and behaviors in the digital environment through behavioral interventions. Many digital self-control tools operate as self-nudges, enabling users to set up choice architectures that protect their goals from competing temptations [29]. Most of these interventions often use a “just-in-time” approach to intervene precisely when overuse is likely to occur [30].

Several apps have been proposed in recent years with varying techniques and degrees of enforcement [31-36]. For instance, the app MyTime offers real-time feedback on time spent across apps and prompts goal reflection, which led to reduced daily use and increased perceived self-regulation in a randomized trial with college students [31]. MindPhone, a smartphone app that interrupts habitual unlocking with intention prompts, was found to increase awareness and reduce habitual use over 14 days, especially among users with initially higher problematic smartphone use scores [36]. One Sec delays app access with a short breathing pause and prompt. A within-subject field study showed that this design significantly reduced Instagram use and increased perceived control without notable user frustration [35].

Despite promising developments, existing digital self-control tools continue to face critical limitations regarding long-term effectiveness and sustained user engagement [37-39]. Many tools rely on overly restrictive mechanisms, such as app blocking, which can undermine users’ sense of autonomy and trigger psychological reactance—ultimately reducing continued use. Others adopt low-friction approaches, such as passive notifications, which are often ignored at the moment of temptation and fail to interrupt habitual use [37,40-42].

A further limitation lies in the lack of customization: most tools do not allow users to align the intervention with their personal goals, definitions of distraction, or preferred self-regulation strategies. A systematic review of 334 digital self-control tools found that few support the nuanced, user-defined boundaries essential for meaningful and sustainable behavior change [38].

Moreover, evidence on the effectiveness of many digital self-control tools remains limited, particularly with regard to addressing the underlying psychological mechanisms of problematic social media use, such as impulsivity, habitual behavior, and deficient self-control [30,39]. Although a wide variety of tools exists, few interventions integrate empirically grounded behavior change strategies with autonomy-supportive design principles. This presents a critical gap in the literature: the need for digital self-control tools that combine customization, user agency, and theory-based mechanisms of change. Studies indicate that users are more likely to engage with interventions that allow them to set individualized goals, determine when and how reminders are delivered, and track their progress toward greater self-regulation [43,44].

This study addresses this gap by evaluating the Wellspent app, a customizable, autonomy-supportive intervention designed to promote self-regulated social media use. By combining evidence-based behavior change techniques (BCTs) [45] with flexible, user-driven customization, Wellspent aims to effectively support users in regaining control over their usage patterns while preserving users’ sense of autonomy. Through a preregistered randomized controlled trial, this study offers one of the first rigorous evaluations of a digital self-control tool specifically designed to target problematic social media use in a personalized and theory-informed manner.

### Aims and Research Questions

The increasing need for effective tools to support healthier and more intentional social media use has led to the development of the Wellspent app, a theory-informed digital self-control tool. The app represents a novel approach to digital self-regulation by combining evidence-based BCTs [45], such as goal setting, behavioral feedback, self-monitoring, and intention-based nudges, with a high degree of user autonomy and customization. Unlike many existing tools that rely on rigid blocking mechanisms or generic notifications [38], the Wellspent app allows users to define their own usage goals, select problematic apps, and personalize reminder timing, tone, and content.

A core differentiator of the Wellspent app lies in its autonomy-supportive design. Rather than enforcing restrictions, the app preserves users’ freedom to choose whether to quit or continue using an app after each prompt. This design is grounded in the dual-process model of behavior regulation [22], aiming to interrupt automatic, habitual engagement (System 1) and foster more reflective, goal-directed decision-making (System 2). Prior research suggests that such personalized and autonomy-supportive interventions may enhance message resonance, reduce psychological reactance, and improve long-term adherence [46]. With its emphasis on autonomy, customization, and theory-driven behavior change, this study addresses a critical gap in the digital well-being literature and contributes to the development of more effective, user-centered self-regulation tools.

The significance of this study lies in its methodological rigor. To date, few interventions in the field of digital self-regulation have undergone a rigorous empirical evaluation using gold-standard methods [47,48]. This study presents a preregistered randomized controlled trial with 70 participants, comparing the effects of the Wellspent app with a no-treatment control group. The design ensures high internal validity and allows for causal inferences regarding the app’s impact on behavior.

An earlier publication from the overarching study project on intervention effects examining separate outcomes found no significant effects on overall well-being, affect, or stress but observed reduced stress among users with initially high levels of problematic smartphone use in the intervention group [47]. The primary objective of this study is to investigate whether participants in the intervention group receiving the Wellspent app, compared to a control group, demonstrate reductions in problematic social media use, problematic smartphone use, and time spent on the most problematic social media app, as well as an increase in self-efficacy for self-regulating social media app use.

## Methods

### Study Design and Participants

This study presents findings from a randomized controlled trial designed to evaluate the effectiveness of the Wellspent app, a digital self-control tool aimed at promoting self-regulated social media use on smartphones. The trial was preregistered at the German Clinical Trial Register (DRKS00031767) on May 8, 2023. The first participant was enrolled on May 22, 2023, and a sample size of 108 participants was targeted. The study was conducted between May and June 2023.

Participants were recruited via private social media channels (Facebook, LinkedIn, WhatsApp, and Instagram) and email lists for study opportunities at the Freie Universität Berlin, Germany. Prospective participants enrolled through an online registration page, where they received information regarding the study’s purpose, data protection guidelines, and the voluntary nature of participation. Informed consent was obtained from all participants prior to their inclusion in the study.

Inclusion criteria required participants to be at least 18 years of age, proficient in English, own an iPhone with iOS 16 (as the Wellspent app was exclusively available for this operating system), and regularly use social media. Social media was broadly defined as platforms enabling users to engage with content, share updates, or interact with others online (eg, Instagram, Facebook, and TikTok).

The final study sample consisted of 70 participants (47/70, 67% female; mean age 26.2, SD 5.6 years), with 35 participants allocated to each group. The remaining 70 participants were randomly assigned (1:1) by the author team to either the intervention group (n=35) or the control group (n=35) using a simple randomization procedure via Research Randomizer [49]. No blocking or stratification was used. Formal blinding was not implemented, as participants were aware of their group assignment.

**Figure 1.**
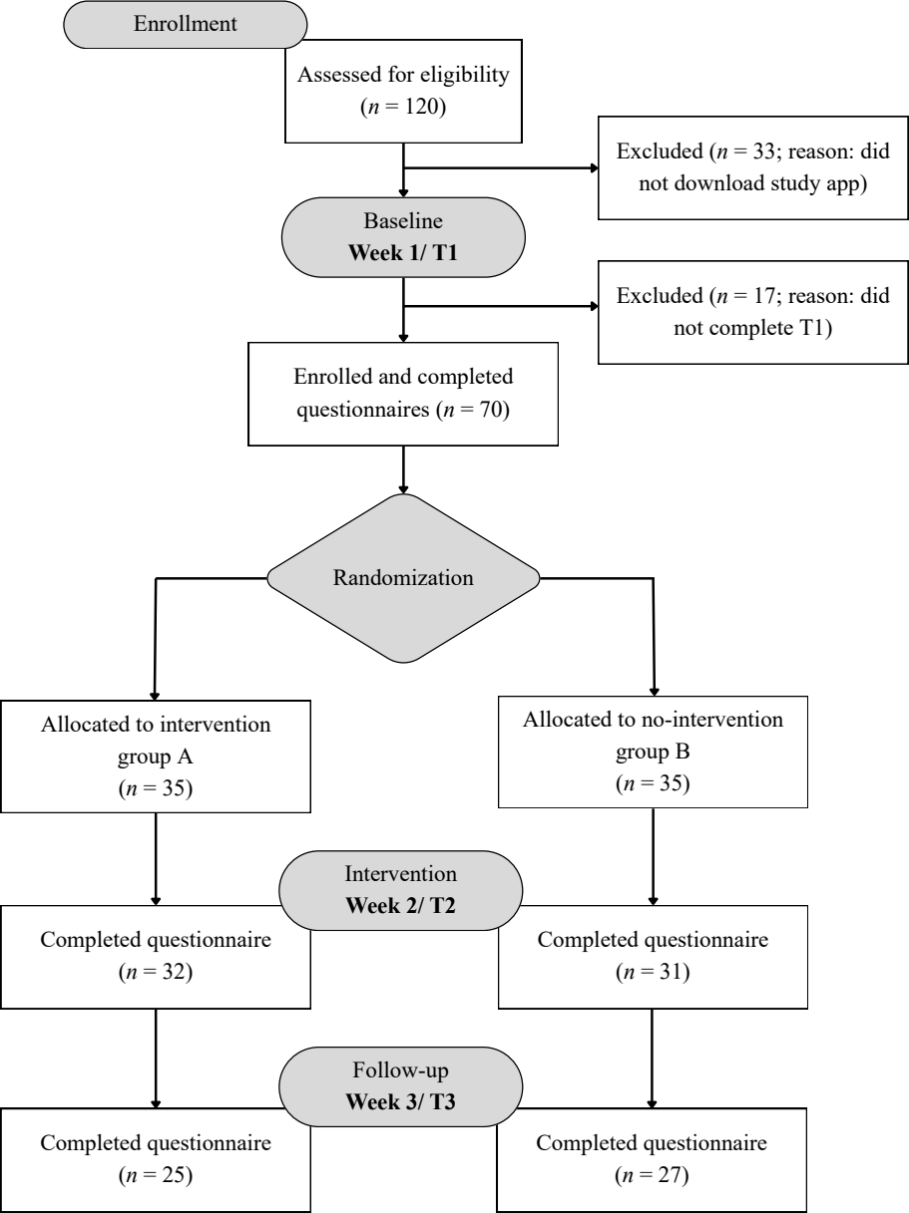
Flow diagram outlining participant allocation into the intervention or control group.

Participants assigned to the intervention group received the app’s intervention features during week 2. The control group received no intervention and continued using their phones as usual. Use of the intervention app in week 3 was voluntary for the intervention group. Both groups completed follow-up assessments at the end of week 2 (T2) and week 3 (T3). At each time point, participants were prompted via email to complete the respective questionnaires, with follow-up reminders sent 24 hours later. Demographic data (including age, sex, level of education, and occupation) were collected at baseline (T1; Figure 1).

### Ethical Considerations

This study was approved by the Ethics Committee of the Department of Education and Psychology at Freie Universität Berlin, Germany (proposal number 009.2023; approval date May 5, 2023). Prior to enrollment, all participants provided informed consent, having received detailed information about the study’s purpose, data processing procedures, and their right to withdraw at any time. To ensure privacy and confidentiality, all data were pseudonymized at the point of data collection, and no personally identifiable information was retained. All collected data were stored on secure servers in accordance with the General Data Protection Regulations regulations. Participants were compensated with academic course credits (when they were students) and received a 1-year premium account for the Wellspent app, provided by the app’s developer; no other financial incentives were offered.

### Intervention

The Wellspent app is a theory-informed, customizable mobile intervention developed to support self-regulated social media use. It targets user-identified problematic apps and delivers personalized behavioral nudges. The intervention draws on BCTs from the BCT Taxonomy v1 [45], addressing psychological mechanisms associated with problematic social media use [50]. Specifically, the intervention incorporates the following BCTs [51]:

1. Goal setting (behavior; Behavior Change Intervention Ontology [BCIO: 007004]): users define daily time limits for selected social media apps.
2. Prompts/cues (BCIO: 050578): full-screen reminders are displayed when usage exceeds a user-defined threshold.
3. Feedback on behavior (BCIO: 007022): real-time messages show time spent on selected apps.
4. Self-monitoring of behavior (BCIO: 007024): visual summaries allow users to track progress toward their individual goals.
5. Substitution of behavior (BCIO: 007095): users specify alternative behaviors to engage in when prompted to take a break from social media.

The intervention was designed according to self-nudging principles [29], enabling users to customize the app’s behavior to align with their habits, goals, and preferences. This approach preserves autonomy and supports reflective (System 2) rather than habitual (System 1) decision-making [22]. Customization features and exemplary reminder nudges are detailed in Multimedia Appendix 1.

**Figure 2.**
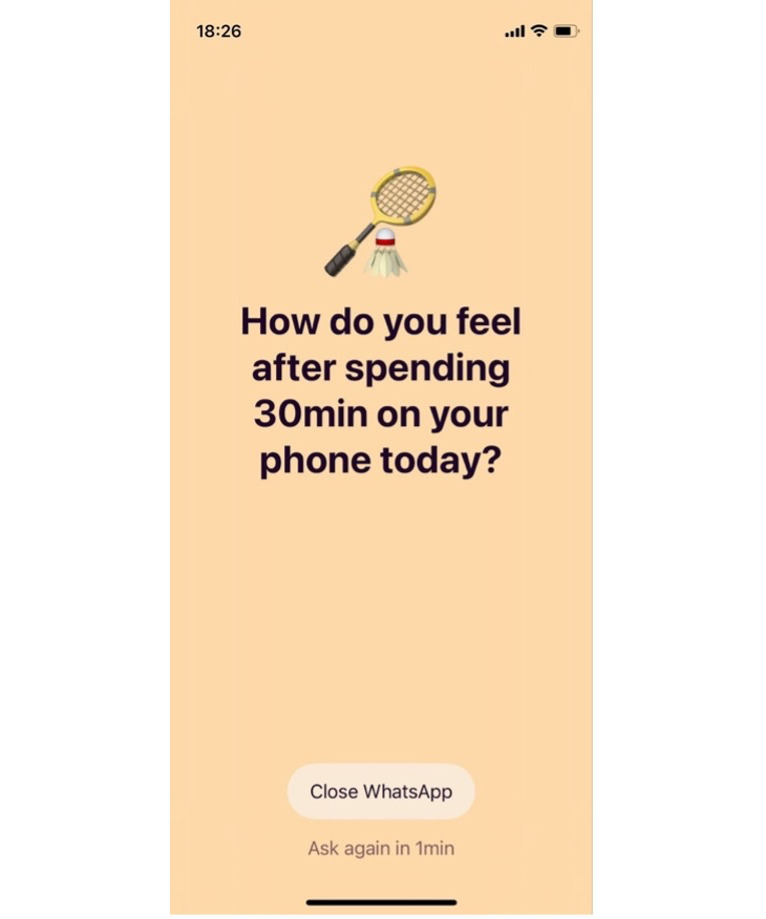
A screenshot of an exemplary reminder message from the Wellspent app.

All users in the intervention group completed the same standardized setup process:

1. Installation and onboarding: participants installed the Wellspent app on their iPhones (iOS 16+).
2. Selection of problematic apps: participants identified one or more social media apps they personally considered problematic (eg, Instagram and TikTok).
3. Goal setting: for each selected app, participants defined a daily usage limit (eg, 45 min) and a nudge interval (eg, after 10 min of continuous use).
4. Customization of reminder prompts: participants tailored full-screen reminders to their preferences:
  -  Reminder frequency: how often reminders should appear once the nudge interval is exceeded (eg, every 3 min).
  - Alternative activity: users selected an activity to engage in instead of scrolling (eg, reading and exercising).
  -  Tone of voice: participants chose the communicative style of the reminder (eg, motivational or reflective). See Figure 2 for an example.
  -  “Danger Zones” (optional): users could define vulnerable time windows (eg, before bed), during which reminders became more salient.
5. Triggering of full-screen reminders: once the self-defined nudge interval was exceeded during app use, a full-screen reminder appeared. These reminders required active dismissal but did not block app access, allowing users to decide whether to continue or take a break.
6. Timing within the study: the intervention began in week 2. In week 3, continued app use remained voluntary for participants in the intervention group.

### Measures

Weekly self-report measures were collected between baseline (T1) and follow-up (T3) to assess the following outcomes: problematic social media app use, problematic smartphone use, self-efficacy to self-regulate social media app use, and participants’ screen time for their most problematic social media app.

#### Problematic Social Media App Use

The primary outcome, problematic social media app use, was measured using an adaptation of the Bergen Social Networking Addiction Scale (BSNAS) [52]. The BSNAS comprises 6 items, such as “How often during the last 7 days have you used social media so much that it has had a negative impact on your job/studies?” rated on a scale from 1 (“very rarely”) to 5 (“very often”). The internal consistency of the baseline problematic social media app use scale demonstrated acceptable reliability (α=.77).

#### Problematic Smartphone Use

Self-reported problematic smartphone use was included as an additional measure beyond preregistered outcomes to examine effects on overall smartphone use and its known correlation with various health outcomes [53]. Problematic smartphone use was measured with a single item: “How often during the 7 last days have you found your smartphone use problematic?” rated on a scale from 1 (“very rarely”) to 5 (“very often”). Prior research indicates a high correlation between this 1-item measure and the established scale from Foerster et al [54] in an earlier study [48].

#### Self-Efficacy to Self-Regulate Social Media App Use

Three items adapted from Keller et al [48] measured self-efficacy to self-regulate social media app use. Participants responded to statements commencing with “During the last 7 days have you felt that you can...” (eg, “...use social media apps consciously even if I first have to find a way to integrate this into my daily routine.”), rated on a scale from 1 (“not at all true”) to 6 (“exactly true”). The internal consistency at baseline (T1) indicated acceptable reliability (α=.63).

#### Social Media App Use

Participants’ daily minutes spent on their most problematic social media app were derived from objectively measured screen time app records. This data was input by participants in hours and minutes for their most problematic social media app when completing the questionnaires for the respective study weeks (week 1, week 2, and week 3). Weekly scores were calculated by averaging the daily minutes of social media app use over the past 7 days. Univariate outliers (*z*>3.29) were winsorized to 1 unit higher than the highest valid value, with *z*<3.29 in the distribution [55].  

### User Acceptance

User acceptance of the intervention was defined as the user refraining from dismissing a reminder (and thereby continuing their app use) within 1 minute of receiving the reminder nudge. The acceptance rate of the Wellspent app’s reminders was computed by dividing the instances of accepted interventions by the total number of occurrences.

### Qualitative Information

Qualitative data were collected through an online survey conducted via Qualtrics at the end of the intervention period. Participants in the intervention group answered a series of 10 open-ended questions aimed at exploring their experiences with the Wellspent app (Table S1 in Multimedia Appendix 2). These questions included prompts such as “How would you describe the Wellspent app to a friend?” and “What does the Wellspent app help you with?” The questions were designed to gather insights into users’ perceptions of the app’s functionality, usability, and effectiveness, as well as to identify potential areas for improvement. A thematic analysis approach was used informally to identify key themes from participants’ responses [56].

### Covariates

The list of covariates included participants’ age, sex (women=1; male=0), education (1=university degree; 0=no university degree), positive affect, and screen time in minutes at baseline. Positive affect was measured with an adaptation of the Positive and Negative Affect Schedule with items such as “Please indicate the extent you have felt this way when thinking about your social media app use over the last 7 days” (1=“very slightly or not at all”; 6=“extremely”) [47].

### Data Preparation and Preliminary Analyses

Prior to hypothesis testing, data were screened and cleaned. Univariate outliers in screen time (*z*>3.29) were winsorized to 1 unit above the highest valid score with *z*<3.29, following recommendations by Tabachnick and Fidell [55]. To assess baseline comparability between the intervention and control groups, *t* tests and chi-square tests were conducted for key demographic and outcome variables. These tests also informed attrition analyses comparing retained and dropout participants.

### Statistical Analysis

#### Power

A power analysis was conducted using G*Power (version 3.1.9.7; Heinrich-Heine-Universität Düsseldorf) [57] to determine the necessary sample size for a repeated measures ANOVA, for 2 groups across 2 measurement points in time (ie, from baseline to postintervention and baseline to follow-up) [58]. It indicated that a minimum of 54 participants (27 per group) were required to identify a significant group × time interaction effect. This calculation assumed a medium effect size of 0.25 (Cohen medium effect size) [35,48,58], with a significance level (α) of .05, and a power value of 1–β=0.95 [59]. Anticipating a 50% dropout rate, as observed in a similar intervention study by Keller and colleagues [48], our target recruitment was set at a minimum of 108 participants.

#### Main Analysis

Data analyses were conducted according to the intention-to-treat approach using both SPSS (version 29; IBM Corp) and RStudio (version 4.3.2; R Core Team) [60]. While SPSS was primarily used for data cleaning purposes, RStudio with its *lme4* package [61] was used for multilevel modeling. We acknowledge that this data analysis deviated from the ANOVA-based power analysis; however, multilevel modeling accounts for the nested data structure and the within-group and between-group variability. Group differences in problematic social media use, problematic smartphone use, self-efficacy, and screen time were assessed using linear mixed models. These models were structured with 3 time points (T1, T2, and T3; within level) nested within participants (between level), and to apply a restricted maximum likelihood procedure in order to account for missing data [61]. To determine intervention effects on the outcome measure, time (0=T1, 1=T2, and 2=T3) × experimental condition (0=control group and 1=intervention group) interactions were estimated as predictors. In subsequent sensitivity analyses, age, sex, positive affect, education level, and screen time in minutes were included as covariates in models 1‐3. Model 4 included age, sex, positive affect, and education level as covariates. While we are unable to share participant data due to privacy regulations, the R code for the data analyses is available in the project’s Open Science Framework repository [62].

## Results

### Sample Characteristics, Randomization, and Attrition Check

Of the 120 persons screened for eligibility, 33 of them did not install the Wellspent app, and 17 persons did not complete the baseline questionnaire. The randomized sample consisted of 70 individuals (47 women and 23 men) with a mean age of 26.19 (SD 5.63; range 18‐45) years. Figure 1 shows a CONSORT (Consolidated Standards of Reporting Trials) flow diagram outlining the study’s design and participant allocation across the randomized controlled trial with 3 measurement points. Most participants were students or young professionals. Participants’ most chosen problematic social media app was Instagram (29/35, 82%), followed by TikTok (4/35, 10%) and Twitter (1/35, 4%). Detailed demographic data are presented in Table 1, and descriptive statistics for all outcome measures across time and condition are provided in Table 2.

**Table 1.** Demographic characteristics of the baseline sample and per study condition. Table reproduced from [47].

Variables	Total sample (n=70)	Intervention group (n=35)	Control group (n=35)
Sex, n (%)			
Female	47 (67)	22 (63)	25 (71)
Male	23 (33)	13 (37)	10 (29)
Age (years), mean (SD)	26.19 (5.63)	26.77 (6.56)	25.6 (4.53)
Nationality, n (%)			
German	56 (80)	28 (80)	28 (80)
American	3 (4)	2 (6)	1 (3)
Other nationalities	11 (16)	5 (14)	6 (17)
Education, n (%)			
Doctorate degree	3 (4)	1 (3)	2 (6)
Masters degree	13 (19)	8 (23)	5 (14)
Bachelor degree	23 (33)	10 (29)	13 (37)
Professional degree	4 (6)	2 (6)	2 (6)
Diploma	1 (1)	1 (3)	0 (0)
Trade	1 (1)	0 (0)	1 (3)
High school graduate	25 (36)	13 (37)	12 (34)
Employment, n (%)			
Student	39 (56)	21 (60)	18 (51)
Full-time	20 (29)	10 (29)	10 (29)
Part-time	8 (11)	2 (6)	6 (17)
Unemployed	1 (1)	1 (3)	0 (0)
Other	2 (3)	1 (3)	1 (3)

**Table 2.** Descriptive statistics of study outcomes for both experimental conditions. Reduced sample size for social media app use is due to missing data.

Variable and time point		n	Intervention group, mean (SD)	n	Control group, mean (SD)
Problematic social media app use (rating scale: 1-5)					
T1^a^		35	2.58 (0.76)	35	2.79 (0.71)
T2^b^		32	2.47 (0.61)	31	2.69 (0.66)
T3^c^		25	2.32 (0.72)	27	2.77 (0.68)
Problematic smartphone use (rating scale: 1-5)					
T1		35	3.00 (0.94)	35	3.03 (0.99)
T2		32	2.50 (0.95)	31	3.16 (0.90)
T3		25	2.68 (0.85)	27	3.37 (0.84)
Self-efficacy to self-regulate smartphone app use (rating scale: 1-6)					
T1		35	3.30 (0.70)	35	3.24 (0.70)
T2		32	4.03 (0.73)	31	3.80 (0.76)
T3		25	3.95 (0.90)	27	4.12 (0.72)
Social media app use (min/d)					
T1		21	77 (62)	24	59 (51)
T2		21	54 (46)	24	59 (44)
T3		21	46 (34)	24	63 (48)

a T1: baseline.

b T2: postintervention.

c T3: follow-up.

Randomization confirmed no significant differences across baseline variables between groups. In the attrition analysis, we found that participants who dropped out from the study before T3 (n=18) showed higher levels of positive affect than participants retained in the longitudinal sample (n=52; nonretainers: mean 2.59, SD 0.53 vs retainers: mean 2.20, SD 0.67; *P*=.03). Among the total sample, a subsample of 52 participants (representing a dropout rate of 26%) completed the study by week 3, comprising 25 participants from the intervention group and 27 participants from the control group. These numbers are almost in accordance with the power analysis, which indicated a targeted longitudinal sample size of 27 persons per group. The attrition rate of the present study aligns with observations from other online-based studies [63]. Table 1 provides an overview of key demographic characteristics of the baseline sample.

### Changes in Study Outcomes Over Time

#### Overview

**Figure 3.**
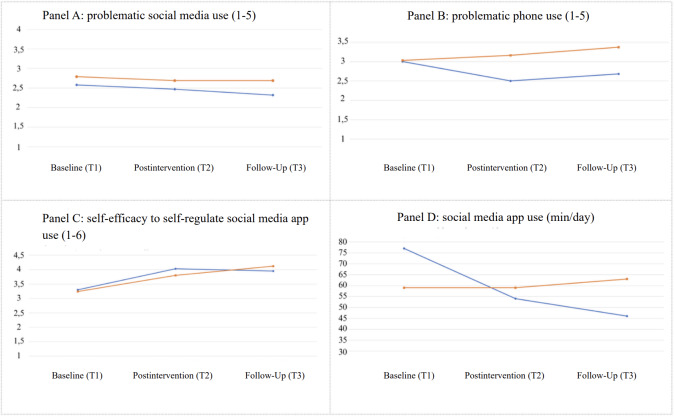
Mean levels over time of outcome variable in the intervention (blue) and control (orange) group. Only parts of the response scales are displayed in panels A, B, and C.

Mean levels of problematic social media app use, problematic smartphone use, social media app use, and self-efficacy to self-regulate social media app use over time and across both conditions were examined and presented in Table 2 and visualized in Figure 3. Within the intervention group, 33% (6430/19,522) of reminder events in week 2 led to participants refraining from further app use within 1 minute after receiving the reminder. This rate increased slightly to 34% (5394/15,996) in week 3. Notably, within the intervention group, 25 (78%) participants voluntarily continued their use of the Wellspent app in week 3.

#### Primary Outcome: Problematic Social Media Use

For the primary outcome, problematic social media app use, the multilevel model revealed a nonsignificant time × group interaction effect (model 1: estimate [Est]=−0.27, SE 0.13, 95% CI −0.51 to –0.01; *P*=.05; intraclass correlation [ICC]=0.69).

#### Secondary Outcome: Problematic Smartphone Use

Problematic smartphone use showed a significant time × group interaction effect (model 2: Est=−0.46, SE 0.18, 95% CI –0.80 to –0.11; *P*=.01; ICC=0.52), indicating improvements in the intervention group when compared to the control group (Figure 3, Panel B).

#### Secondary Outcome: Self-Efficacy to Self-Regulate Social Media Use

Self-efficacy to self-regulate social media use showed a similar increase over time in both the intervention and control group (model 3; time effect: Est=0.57, SE 0.11, 95% CI 0.34-0.78; *P*<.001; ICC=0.34), but no significant time × group effect emerged (model 3: Est=−0.10, SE 0.17, 95% CI −0.44 to 0.22; *P*=.55).

#### Secondary Outcome: Social Media App Use

Regarding time of social media app use, a significant time × group effect was observed (model 4: Est=−29.35, SE 6.84, 95% CI −42.79 to −15.99; *P*<.001). This indicates that social media app use significantly decreased in the intervention group (vs the control group), with an average reduction of approximately 29 minutes per day from week 1 to week 3 (Figure 3, Panel D). The overall pattern of results remained consistent when covariates age, sex, positive affect, and education level were added in sensitivity analyses (reported in Table 3). For problematic social media use, the pattern differed, with the effect reaching statistical significance only in the adjusted model. This suggests that the finding may not represent a robust effect.

**Table 3.** Multilevel model estimates of outcome variables up to a 3-week follow-up. SEs are reported for fixed effects. Random effects are summarized as SDs with 95% CIs.

	Model 1: problematic social media app use			Model 2: problematic smartphone use			Model 3: self-efficacy to self-regulate social media app use			Model 4: time of social media app use		
Fixed effects	Est^a^ (SE)	*P* value	95% CI	Est (SE)	*P* value	95% CI	Est (SE)	*P* value	95% CI	Est (SE)	*P* value	95% CI
Intercept at baseline	2.43 (0.21)	<.001	2.04 to 2.82	2.47 (0.30)	<.001	1.91 to 3.03	3.47 (0.25)	<.001	2.98 to 3.97	78.27 (14.51)	<.001	49.37 to 106.26
Time	0.02 (0.09)	.80	–0.15 to 0.19	0.13 (0.12)	.27	–0.10 to 0.37	0.57 (0.11)	<.001	0.34 to 0.78	3.70 (4.53)	.40	–5.14 to 12.60
Group	–0.08 (0.18)	.67	–0.41 to 0.26	–0.10 (0.26)	.71	–0.58 to 0.39	0.12 (0.20)	.57	–0.30 to 0.50	22.79 (13.86)	.10	–3.72 to 49.33
Group by time	–0.27 (0.13)	.05	–0.51 to –0.01	–0.46 (0.18)	.01	–0.80 to –0.11	–0.10 (0.17)	.55	–0.44 to 0.22	–29.35 (6.84)	<.001	–42.79 to –15.99
Age	–0.09 (0.02)	<.001	–0.13 to 0.04	–0.10 (0.03)	.01	–0.17 to –0.04	0.05 (0.03)	.09	–0.01 to 0.11	–2.30 (1.60)	.16	–5.30 to 0.69
Sex^b^	0.21 (0.16)	.20	0.09 to 0.51	0.35 (0.26)	.07	–0.001 to 0.86	0.15 (0.20)	.47	–0.28 to 0.55	1.50 (10.73)	.89	–19.47 to 22.40
Education^c^	0.03 (0.18)	.86	–0.29 to 0.36	0.35 (0.26)	.18	–0.12 to 0.82	–0.34 (0.22)	.13	–0.78 to 0.08	–37.61 (11.79)	<.001	–60.63 to –14.65
Phone usage	–0.00 (0.00)	.62	–.001 to .001	–0.00 (0.00)	.69	–0.001 to 0.001	–0.00 (0.00)	.09	–0.001 to 0.00	—^d^	—	—
Positive affect	0.10 (0.12)	.40	–0.12 to 0.32	–0.15 (0.17)	.37	–0.46 to 0.16	–0.28 (0.14)	.06	–0.56 to‐0.03	–3.69 (7.77)	.64	–19.15 to 11.82
Random effects (SD)												
Intercept	0.50	-—	0.31 to 0.62	0.64	—	0.32 to 0.83	0.40	—	0.18 to 0.64	42.77	—	31.13 to 52.03
Time	0.29	—	0.00 to 0.42	0.19	—	0.00 to 0.49	0.14	—	0.01 to 0.46	16.42	—	6.18 to 23.21
Residual variance	0.34	—	0.28 to 0.42	0.61	—	0.50 to 0.72	0.59	—	0.46 to 0.67	16.61	—	13.61 to 20.89
ICC^e^	0.69	—	—	0.52	—	—	0.34	—	—	0.87	—	—
Pseudo-*R*^2^	0.78	—	—	0.62	—	—	0.57	—	—	0.88	—	—

a Est: estimate.

b Sex coded as 0=men and 1=women.

c Education coded as 0=no tertiary education and 1=tertiary education.

d Not applicable.

e ICC: intraclass correlation.

### Qualitative Results

Qualitative feedback was explored informally by reviewing the open-ended responses from the intervention group, collected via Qualtrics at the end of the intervention period. Responses were grouped into recurring patterns based on the researcher’s judgment, without following a formal coding or analysis protocol. Emerging themes included the following.

#### App Effectiveness and Positive Feedback

Participants provided positive feedback on the app’s effectiveness in managing screen time, frequently praising its simplicity and user-friendly interface. One participant remarked, “It’s a very easy-to-use and straightforward app that allows you to monitor your social media use through reminders sent during self-chosen time intervals.” (P26). The app’s reminders were particularly appreciated for interrupting mindless scrolling, serving as timely prompts about time spent on social media, and encouraging users to put their phones down. For instance, a participant noted, “Great app that keeps you alarmed that there’s life outside of the social media apps.” (P32). The ability to set individual budgets for different apps was also considered valuable for promoting conscious social media use: *“*The budgets are very useful because different apps need different kinds of monitoring.” (P26).

#### Concerns and Areas for Improvement

The main concerns raised by the intervention group primarily revolved around technical bugs, such as repeated reminders after dismissal, inaccuracies in screen time tracking, and restrictions on selected apps. These issues were seen as areas for improvement, as they impacted the overall user experience.

#### Integration Into Daily Life and User Acceptance

Participants expressed varying views on integrating the Wellspent app into their daily social media use. Some highlighted the app’s value in enhancing awareness and reducing usage time, describing it as “helpful but not intrusive” (P21). Others underscored its assistance in enabling alternative activities: “It helps me to do other things instead.” (P10). However, instances emerged where user acceptance was hindered, particularly when the app’s reminders interrupted enjoyable social media experiences: “It can be annoying when you are enjoying being on social media, and have done so in moderation, to get constant notifications. I ended up moving the nudge interval when I wanted to use social media for longer periods of time.” (P3).

Overall, while participants generally acknowledged the app’s efficacy in monitoring screen time, some expressed a need for improvements in certain features to fully embrace its integration into daily life. Suggested enhancements included additional statistics tracking (eg, weekly screen time reduction), enhanced friction when exceeding limits (eg, app lock with a code), different modes (eg, study, work, or intentional scrolling mode), and adaptive nudge intervals (eg, prolonged nudge intervals after extended periods of nonuse).

## Discussion

### Principal Results

This randomized controlled trial investigated the effectiveness of the customizable intervention app Wellspent in supporting self-regulated social media use. The intervention produced significant reductions in daily time spent on the most problematic app (approximately 29 minutes) and reduced perceived problematic smartphone use. In contrast, no significant effects were found for problematic social media use or self-efficacy to self-regulate social media use. These findings suggest that behavioral change, in the form of reduced usage time, can occur relatively quickly through moderate, autonomy-supportive interventions, whereas cognitive and self-perception outcomes may require longer, more reflective processes.

### Effects on Problematic Social Media Use

The intervention did not significantly decrease problematic social media use. This aligns with research showing that problematic use arises from deeper, multifaceted patterns, such as emotional health [16], habit strength [64], and personality characteristics [65], making it difficult to change quickly. The intervention app may not have supplied the sustained cognitive scaffolding needed to alter participants’ perceived problematic use within 2 weeks.

### Effects on Problematic Smartphone Use

The intervention group reported lower levels of problematic smartphone use compared to the control group. This suggests that behavioral reductions may have translated into a more general feeling of control over one’s device. Notably, perceived problematic smartphone use may be easier to shift in the short term than problematic social media use because it reflects global awareness (“I use my phone too much”) rather than app-specific patterns linked to social, emotional, or identity-related motives.

### Effects on Self-Efficacy to Self-Regulate Social Media App Use

Self-efficacy significantly increased in both groups, but participants from the intervention group did not exceed those from the control group. Self-efficacy typically grows through repeated mastery experiences and feedback on successful behavior regulation [66]. As the intervention app focused on in-the-moment friction rather than visible progress tracking, participants may have lacked the experience of sustained success and explicit feedback loops. Similar interventions have also failed to find strong between-group intervention effects on self-efficacy [19], pointing to the need for longer interventions, richer in feedback and reflection.

### Effects on Social Media App Use

The intervention produced a reduction in time spent using social media apps of approximately 29 minutes per day. This finding aligns with prior research highlighting the potential of digital self-control tools to reduce usage time when users are nudged at critical moments [35,40]. Compared to digital self-control tools with generic interventions or one-size-fits-all designs [38], Wellspent offered a tailored and user-centric alternative. The full-screen reminder appearing after a personally defined nudge interval (eg, 10 min on Instagram) created a structured moment of pause, allowing users to shift from automatic to deliberate decision-making [22]. Previous literature emphasizes that user autonomy and personalization increase intervention acceptance and long-term engagement [46]. Our results support this perspective, particularly the positive user feedback from intervention participants regarding flexibility and customization. By integrating customizable, goal-aligned nudges into real-time usage patterns, the app sets a precedent for user-centered intervention strategies in digital self-control.

### Limitations and Future Research

Together, the findings support emerging evidence that autonomy-preserving tools can meaningfully influence digital behavior within short periods. These findings highlight three key considerations for practitioners and designers of digital self-control tools:

1. Moderate enforcement can be effective: interventions do not need to be coercive to reduce screen time. Full-screen reminders that offer users a choice, rather than restricting access, can facilitate reflective engagement without triggering reactance.
2. Customization enhances acceptance: giving users the ability to select their target apps, usage limits, and reminder style appears to foster a sense of ownership and autonomy, which in turn increases voluntary engagement and retention. Future digital self-control tools should prioritize configurability to accommodate individual usage patterns and values.
3. Behavioral change may require more than just friction: while nudging can reduce immediate app use, deeper cognitive outcomes such as self-efficacy or problematic social media use may require additional components, for example, reflective journaling, feedback on progress, or social accountability mechanisms.

This study has several limitations. First, the sample size was relatively small, and self-report measures may be subject to bias, particularly regarding subjective app usage and problematic use. The target sample size of 54 participants, as determined by the power analysis, was not reached. The final analysis sample for the multilevel models on the primary outcome consisted of 46 participants, which may have limited the statistical power to detect medium effects. Second, the short intervention duration may have limited the app’s impact on self-efficacy and habitual behavior. Third, although self-efficacy to self-regulate social media use was assessed, a direct measure of self-regulation (eg, action control) was not included in the study. This omission may limit the ability to fully capture the psychological mechanisms underlying digital behavior change. Future studies should incorporate validated measures of self-regulation to provide a more comprehensive understanding of intervention effects. Fourth, the study was limited to iOS users, excluding other relevant target groups.

Future research should explore the long-term effects of customizable digital self-control tools and investigate how additional features, such as social comparison, adaptive feedback, or intention tracking, could enhance both behavioral and psychological outcomes. Mixed method designs integrating behavioral data, qualitative feedback, and longitudinal tracking could provide deeper insights into how users engage with interventions over time.

### Conclusions

This study provides initial evidence for the effectiveness of a customizable, moderately enforced digital self-control tool in reducing social media screen time and perceived problematic smartphone use. By allowing users to define when, where, and how interventions occur, the Wellspent app demonstrates a user-centered approach to digital self-regulation. These findings support the growing consensus that flexible and autonomy-supportive digital self-control tools offer a promising path toward sustainable behavior change in digital environments.

## Supplementary material

10.2196/56824 Multimedia Appendix 1 Screenshots from the intervention app.

10.2196/56824 Multimedia Appendix 2 Qualitative questions at postintervention and follow-up.

10.2196/56824 Checklist 1 CONSORT-eHEALTH checklist (V 1.6.1).

## Abbreviations

BCIO: Behavior Change Intervention Ontology
BCT: behavior change technique
BSNAS: Bergen Social Networking Addiction Scale
CONSORT: Consolidated Standards of Reporting Trials
Est: estimate
ICC: intraclass correlation
IM³UNE model: Integrative Model of Mobile Media Use and Need Experiences
