# Supplementary material for: Promoting Self-Regulated Social Media Use on Smartphones With a Mobile Intervention App (Wellspent): Randomized Controlled Trial
Source: JMIR Mhealth Uhealth. 2026 Apr 8;14:e56824. doi: 10.2196/56824 (PMC13062480; doi:10.2196/56824)
Supplement: Multimedia Appendix 1 [file mhealth-v14-e56824-s001.pdf]

Welcome to the Wellspent study!

We're excited to have you onboard.
To get started, you'll need to enter the unique code that we shared with you.

Please enter your 6-digit code

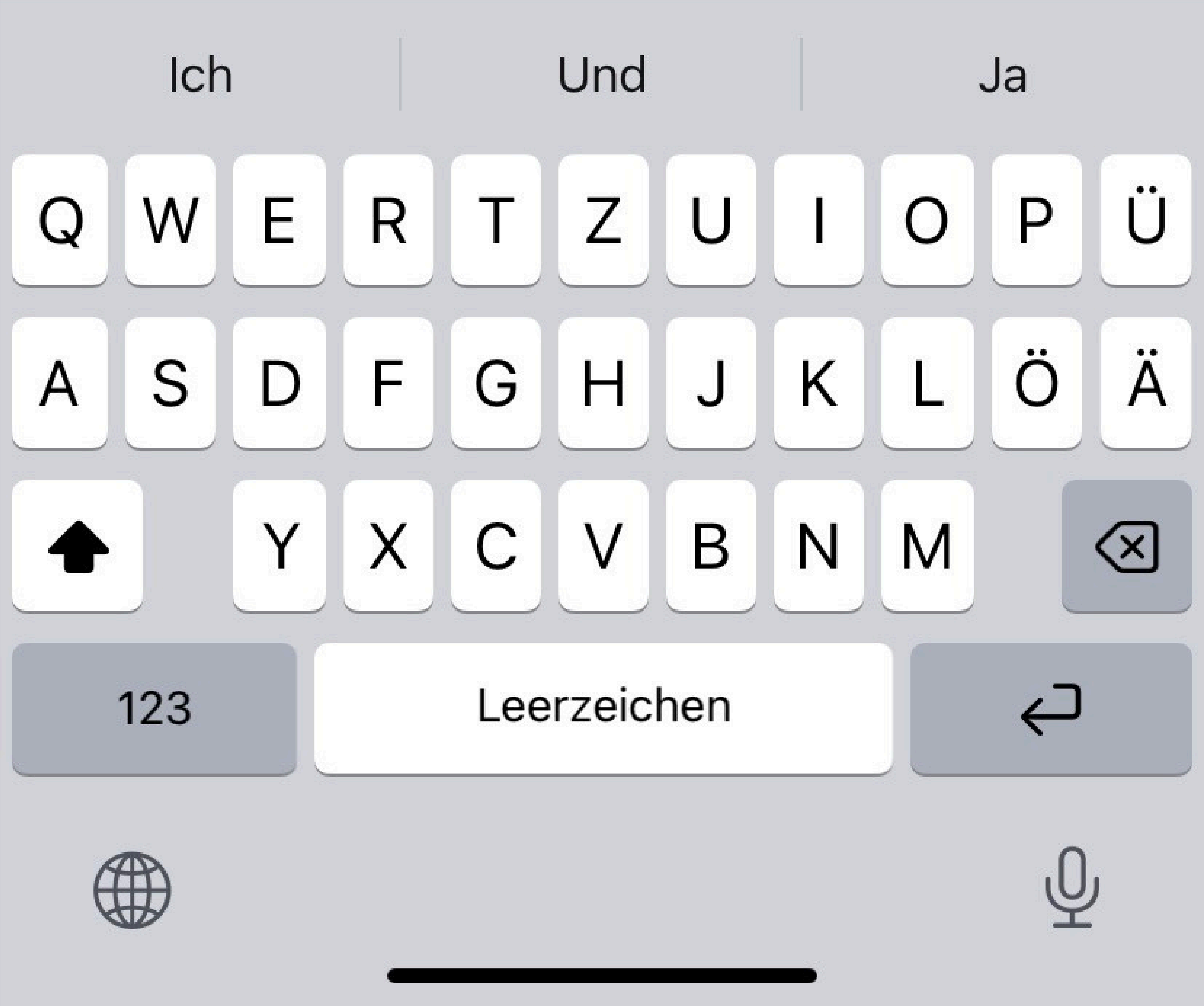

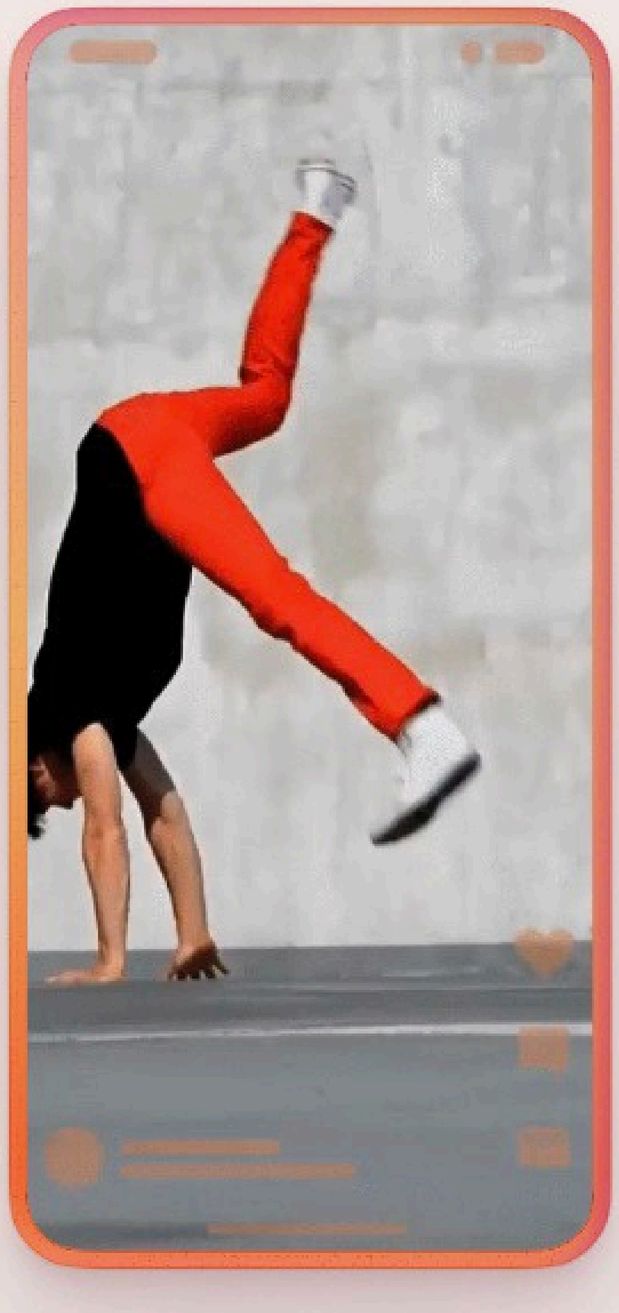

Stop scrolling start living.

Get Started

Terms and Conditions

Privacy Policy

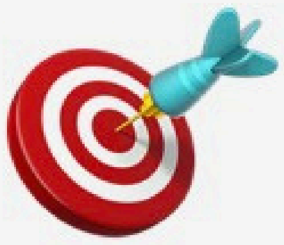

Set your first budget

Decide which Apps or Categories you want to give a time limit.

You can add more budgets later.

Set budget

🔓

Allow Screen Time access

Wellspent needs to see how you use your phone to work properly.

- 🔍 Recognize when you're using distracting apps
- ⌛ Remind you to take breaks or do something else
- 🙋 Tell you when you've reached your budget

Manage Screen Time

What do you want to call this budget?

Make it memorable so you can recognize this budget later (eg. Social Media).

EMOJI **NAME**

👀 Social

Next

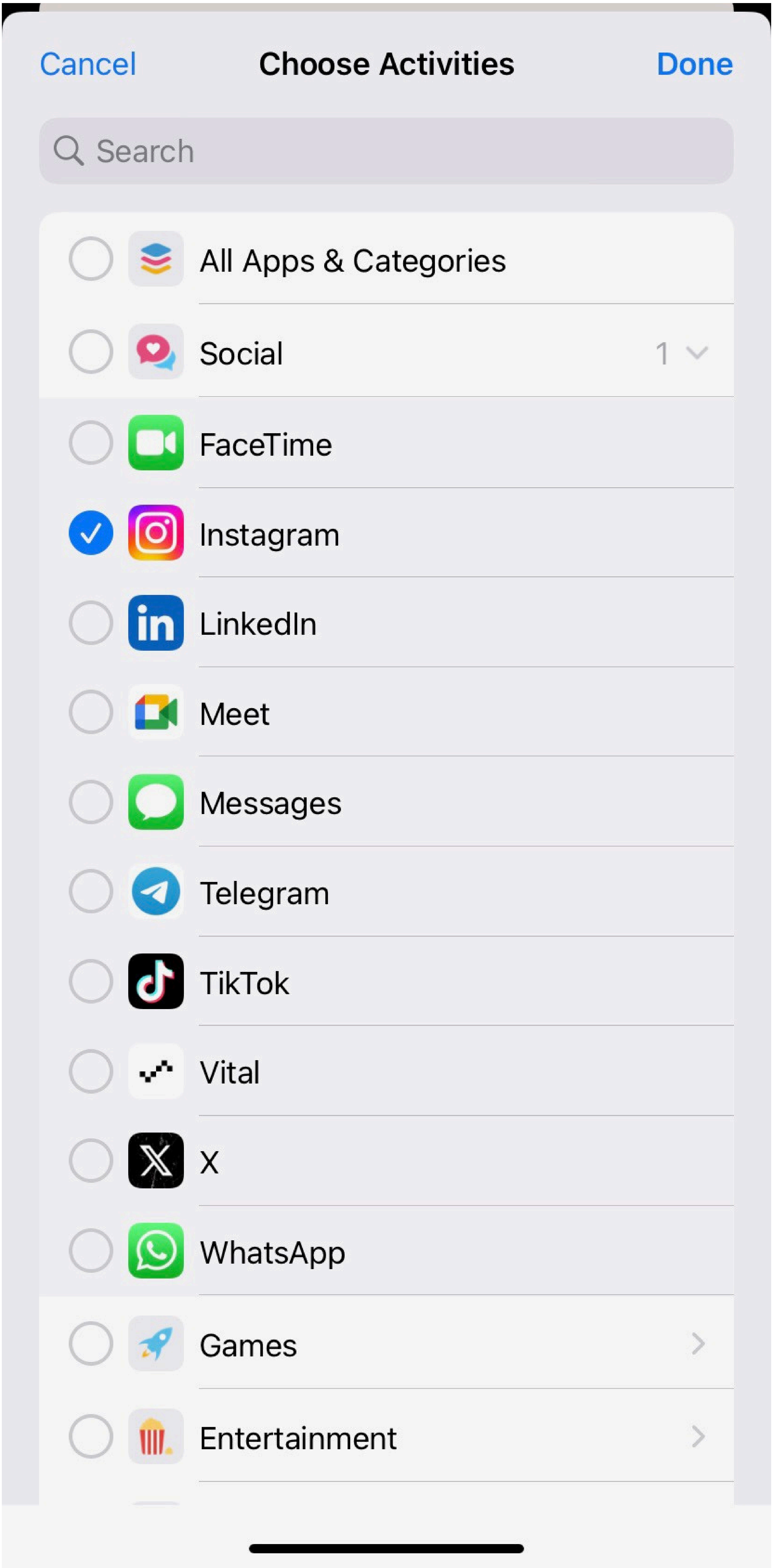
Cancel
Choose Activities
Done
Search
All Apps & Categories
Social
1
FaceTime
Instagram
LinkedIn
Meet
Messages
Telegram
TikTok
Vital
X
WhatsApp
Games
Entertainment

How much time per day do you want to spend on all these apps?

All apps & websites combined that you just selected.
Make it achievable, you can adjust later.

Daily budget 0 hours 30 min

27
28
29
0 hours 30 min
1 31
2 32
3 33

Commit

How much is typical?

< Back

Edit Budget

NAME

Social

Edit

TikTok

Instagram

LinkedIn

Daily budget 0 hours 30 min

This is how much time you want to spend on these apps per day.

Nudge interval 0 hours 7 min

This is how much time you want to spend on these apps before being nudged.

Save settings

Remove goal

Your desired habits

- 📖 Reading ✓
- 💪 Exercising ○
- 🙃 Stretching ○
- 🧘 Mindfulness practice ✓
- 💻 Working ○
- 😴 Sleeping ○
- 🧹 Housekeeping ○

Done

< Profile

Tone of voice

How Wellspent will speak to you

💭 **Reflective**
Wise mentor guiding your reflections

🤗 **Friendly**
Supportive friend urging you on

😈 **Roast** ✓
Witty comedian teasing you into action

🤡 **Fun**
Playful friend making you smile

👏 **Hype** ✓
Energetic cheerleader inspiring action

☝️ **Authoritative**
Tough coach pushing to succeed

Done

Profile Goals

Your danger zones

- ⏳ I get distracted by my phone during work hours ○
- 😵‍💫 I get lost scrolling before going to bed ✓
- 🐓 I'm on my phone first thing in the morning ○
- 🤹 I waste my free-time on my phone ○
- 🧑‍💻 I get lost in social media ○

Knowing about your personal danger zones helps Wellspent to be extra careful during these moments and offer you the support you need.

Done

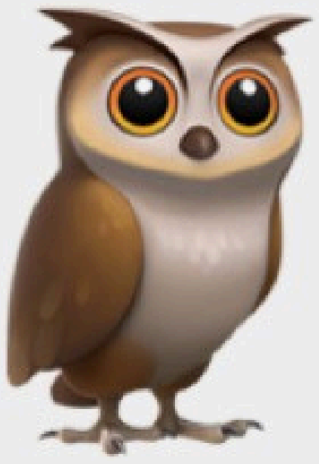

Remember to give your eyes a break from the screen.

You've been on here for 30min.

Close Instagram

Ask again in 1min

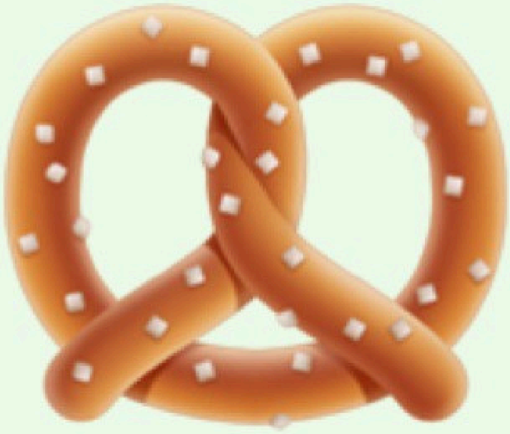

Feeling like a pretzel?

Stretch it out and undo all those twists and turns!

Close Slack

Ask again in 5min

How do you feel after spending 31min on your phone today?

Close WhatsApp

Ask again in 5min
