# Supplementary material for: Promoting Self-Regulated Social Media Use on Smartphones With a Mobile Intervention App (Wellspent): Randomized Controlled Trial
Source: JMIR Mhealth Uhealth. 2026 Apr 8;14:e56824. doi: 10.2196/56824 (PMC13062480; doi:10.2196/56824)
Supplement: Multimedia Appendix 2 [file mhealth-v14-e56824-s002.docx]

# **Multimedia Appendix 2**

Table S1. Qualitative Questions at Post-Intervention and Follow-Up

| **Time Point** | **Question** |
| --- | --- |
| Post-Intervention | How would you describe the Wellspent app to a friend? |
|  | What does the Wellspent app help you with? |
|  | What is the best thing about the Wellspent app? |
|  | What is the worst thing about the Wellspent app? |
|  | What are your thoughts on the Wellspent app’s customization features (i.e., setting up budgets with specific apps/daily limits/nudge intervals and customizing the intervention content by specifying goals/desired habits/tones of voice)? |
|  | Would you integrate the Wellspent app into your daily social media use? Why? |
|  | If you were given a chance to redesign the Wellspent app, what would you change or add to manage your social media use? |
|  | Did you encounter any specific errors or technical bugs? (If yes, please name them.) |
|  | Any additional comments? |
| Follow-Up | Did you continue using the Wellspent app during the last seven days? Why? |
